# Combining Associations Between Emotional Intelligence, Work Motivation, and Organizational Justice With Counterproductive Work Behavior: A Profile Analysis via Multidimensional Scaling (PAMS) Approach

**DOI:** 10.3389/fpsyg.2020.00851

**Published:** 2020-05-19

**Authors:** Aharon Tziner, Erich C. Fein, Se-Kang Kim, Cristinel Vasiliu, Or Shkoler

**Affiliations:** ^1^Peres Academic Center, Rehovot, Israel; ^2^Netanya Academic College, Netanya, Israel; ^3^School of Psychology and Counselling, University of Southern Queensland, Toowoomba, QLD, Australia; ^4^Department of Psychology, Fordham University, New York, NY, United States; ^5^Academia de Studii Economice din Bucuresti, Bucharest, Romania

**Keywords:** emotional intelligence, counterproductive work behavior, organizational deviance, profile analysis, multidimensional scaling, emotional exhaustion, job satisfaction, organizational justice

## Abstract

The need for better incorporation of the construct emotional intelligence (EI) into counterproductive work behavior (CWB) research may be achieved via a unified conceptual framework. Accordingly, the purpose of this paper is to use the Profile Analysis via Multidimensional Scaling (PAMS) approach, and a conceptual framework that unifies motivational process with antecedents and outcomes, to assess differences in EI concerning a variety of constructs: organizational justice, CWB, emotional exhaustion, job satisfaction, and intrinsic motivation. Employing established scales within a framework unifying CWB, intrinsic motivation, EI, organizational justice, and outcome constructs, two EI-based profiles displayed associations with CWB based on responses from 3,293 employees. Both the first core profile, *high overall justice and low emotional intelligence*, and the second core profile, *high emotional intelligence and low work motivation*, displayed associations with interpersonal deviance and organizational deviance, as well as emotional exhaustion and job satisfaction. The results are discussed with respect to possible underlying theory and an overarching unified motivation framework that incorporates goal choice, intrinsic motivation, antecedents, and outcomes. We also provide directions for future research and implications for managers in the workplace based on heuristic conceptual frameworks that combine multiple motivational perspectives into a unified model.

## Introduction

A current gap in the literature concerning counterproductive work behavior (CWB) is how to incorporate an increased range of individual differences, including emotional intelligence (EI), into the network of associations surrounding CWB (Penney and Spector, 2005; Bolton et al., 2010; Ones, 2018). This gap is significant because of the recognized role of individual differences in the overall nomological network that underpins the motivation of behavior at work (Yau and Sculli, 1990; Colquitt et al., 2011; Budnick et al., 2020). For example, EI has been shown to be a critical antecedent of work outcomes (Kashif et al., 2017; Klein et al., 2020), and a critical mediating factor for emotional regulation (Newman et al., 2010; Cheung and Tang, 2012). In addition, as CWB has continued to generate extensive research in the organizational literature, failure in emotional regulation has been increasingly traced to associations with CWB (Bragg and Bowling, 2018).

Understanding EI and its associations with emotional regulation and CWB is critical for managers when staffing and assessing personnel because they are related to essential organizational outcomes such as work quality (Bragg and Bowling, 2018). However, to comprehend fully the context in which EI operates, it is necessary to use networks of attitudes and personal states such as the experience of meaningful work (Simonet and Castille, 2020). In essence, researchers must bridge conceptual frameworks at separate levels by examining goal choice and goal-striving and engagement in conjunction with models of personality and contextual antecedents (Tisu et al., 2020). Within the research presented in this paper, we focus accordingly on connections between EI, CWB, and a delimited, parsimonious set of attitudes, namely, perceptions of organizational justice and job satisfaction, and the dynamic personal states of leader-member exchange (LMX), work motivation, and emotional exhaustion. These attitudes and personal states have been shown to explain consistently large amounts of variability in critical work outcomes such as turnover (Wright and Cropanzano, 1998; Bernerth and Walker, 2012); job performance (Wang et al., 2010), and burnout (Faragher et al., 2013).

Therefore, it would be important, for example, for staffing managers to realize that perceived organizational injustice is a key driver of workplace misbehavior (Everton et al., 2007) and that this effect may be enhanced by incorporating the particularly relevant individual difference construct of EI. We might further note the centrality of perceptions of fairness and justice to well-being at work (Johnston et al., 2016) and the strong and persistent meta-analytic evidence of the predominant contribution of justice as a critical antecedent to job satisfaction and performance (Cohen-Charash and Spector, 2001; Viswesvaran and Ones, 2002; Tziner et al., 2011).

Accordingly, it is important that future research goes further in examining work context variability, and differences in contextually related perceptions, as significant influences in critical work outcomes. For example, despite a general consensus in the literature that indicates that there is generally a consistent, negative relationship between EI and CWB (Dalal, 2005; Miao et al., 2017), the stability of this relationship bears further consideration because nuanced contextual factors can change the strength of this relationship. For example, the effect of EI on OCB is stronger in settings that require and likely habituate employees to engage in emotional labor, such as service and health care settings (Miao et al., 2017).

It would be appropriate, therefore, to use workplace sensitivities, such as justice perceptions, to help explain variations in relationships between traits such as EI and processes such as CWB, with the help of frameworks uniting individual and contextual differences with types of motivation and goal constructs and relevant work outcomes.

The need for better incorporation of the construct emotional intelligence (EI) into CWB research may be achieved via a unified conceptual framework. Accordingly, the purpose of this paper is to use the Profile Analysis via Multidimensional Scaling (PAMS) approach, a conceptual framework that unifies motivational process with antecedents and outcomes, to assess differences in EI concerning a variety of constructs: organizational justice, CWB, emotional exhaustion, job satisfaction, and intrinsic motivation. Within a framework unifying CWB, intrinsic motivation, EI, organizational justice, and outcome constructs and employing established scales, two EI-based profiles displayed associations with CWB based on responses from 3,293 employees.

However, what is often lacking in the organizational psychology literature is research conducted within a unified conceptual framework that connects goal choice and goal-striving perspectives to intrinsic work motivation and personal and contextual-level antecedents. This lack relates to a critical problem within the work motivation literature, which is that motivational phenomena are studied within multiple theoretical frameworks (e.g., goal choice, goal-striving) that are generally operationalized in isolation (Van den Broeck et al., 2019). Consequently, in this paper, we employ a recent integrative motivational framework (Van den Broeck et al., 2019) as a heuristic to understand and operationalize interrelations between goal content, intrinsic motivation, personal and contextual-level antecedents, and important work outcomes. Employing this framework, we model (a) CWB within *goal choice processes* and intrinsic motivation within *goal-striving processes*, (b) organizational justice and EI as *antecedents to goal content and intrinsic motivation*, and (c) emotional exhaustion and job satisfaction as *outcomes*.

**FIGURE 1:**
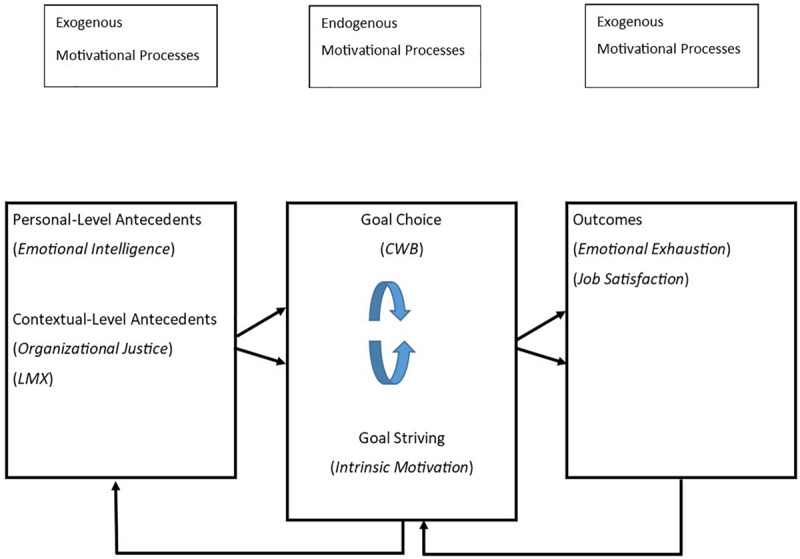
Conceptual framework unifying goal choice and goal striving with personal and contextual antecedents and outcomes. Figure based on a complex model from Van den Broeck et al. (2019).

Specifically, the central purpose of this research is to investigate how (a) a representative individual difference (EI), (b) two critical contextually related perception variables (organizational justice and LMX), and (c) a contextually related motivational state (intrinsic motivation) can model differences in (d) CWB and (e1) a core positive work outcome (job satisfaction) and (e2) a core negative work outcome (emotional exhaustion). Thus, using EI, organizational justice, LMX, and intrinsic motivation, as input variables, we wished to examine associations with CWB, job satisfaction, and emotional exhaustion. As the key organizing framework for our study, we present in Figure 1, an adaption of the unified conceptual framework of Van den Broeck et al. (2019), which is centered on goal choice and goal content.

*Goal choice* describes the selection of one or more goals with a particular type of content that is valued by the individual, along with the selection of the person’s higher-level objectives within personal hierarchies that reflect personal values based on the achievement of complex and long-range goals (Van den Broeck et al., 2019). At lower levels of personal hierarchies, multiple action-oriented objectives serve to advance the achievement of the higher-level goals. For example, a person may have set a goal of increasing feelings of mastery by applying newly acquired knowledge and skill in the workplace. However, to accomplish this goal, the individual must first accomplish the lower-level goal of acquiring supervisor support for training transfer (Zumrah et al., 2012).

The term *goal striving* refers to the individual allocation of cognitive and behavioral effort toward achieving goals within personal hierarchies. With respect to higher-level goals, individuals often strive to achieve these goals through lower-level goal attainment, such as in the above example of acquiring supervisor support for training transfer. Accordingly, goal choice and goal striving are phenomena embedded within hierarchies of goals, and these concepts are modeled within the endogenous motivational processes section of Figure 1, adapted from Van den Broeck et al. (2019).

In the next section, we articulate the importance of four critical constructs – EI, work motivation, organizational justice, and CWB – that are focal to our investigation. Additionally, we highlight research pertinent to the related constructs of LMX, job satisfaction, and emotional exhaustion.

## Critical Constructs

### Emotional Intelligence

Personality traits and individual differences in ability, such as EI, can have important associations with organizational stressors and CWB (e.g., Bowling and Eschleman, 2010; Dixit and Singh, 2019). In addition, researchers have demonstrated that levels of EI in key staff are significant personal factors related to the success and productivity of organizations (e.g., Newman et al., 2010; Karimi et al., 2020). In that context, EI may be defined as the ability to recognize and also monitor one’s own and other people’s emotions, to understand feelings, and subsequently to use emotional information to guide thinking and adapt behavior to suit the environment (Furnham and Taylor, 2020; Robinson et al., 2020).

Regulation of the emotions helps employees to maintain “positive affect,” a positive outlook that influences work behaviors favorably (Newman et al., 2010); additionally, the regulation also restrains “negative affect” (Cheung and Tang, 2012). Hence, employees with high EI have the tools to regulate their emotions and to cope with adversities, and they tend to create emotional and behavioral balance by utilizing self-control and self-regulation (Mayer et al., 2008). However, because EI incorporates both trait and state-based characteristics, we use EI as a critical component within the PASM model.

Related to the study of EI and emotional regulation, researchers suggest there are numerous opportunities for integrating justice, CWB, and job performance through the integration of social exchange, affect states and processes, and emotional regulation (Colquitt et al., 2013). In general, negative affect shows negative associations with justice, while positive affect shows positive associations with justice (Colquitt et al., 2013). Based on these meta-analytic relationships, a clear direction for future research is to test the roles of positive and negative affect and emotional regulation as mediators between justice and performance, and justice and CWB (Colquitt et al., 2013).

At work, individuals with high EI often experience a high level of control, low levels of stress, and high levels of satisfaction and commitment to their work (Petrides and Furnham, 2006). High-EI individuals are also less prone to emotional exhaustion and burnout and are more likely to perform their jobs successfully. In the light of these positive attributes, and the associations articulated in Figure 1, we suggest that emotional intelligence also relieves frustration. Thus, for example, when employees are faced with demotivating factors such as perceived injustice and symptoms of burnout, high-EI employees are less likely to turn to work misbehaviors. Furthermore, based on the integrative framework articulated in Figure 1, we expect that EI will also relate to higher positive work outcomes such as job satisfaction. This anticipated association accords with research that indicates that leaders’ EI is significantly and positively associated with subordinate’s jobs, and that suggests that high EI leaders can serve as “mood managers” within organizations (Miao et al., 2016).

### Intrinsic Work Motivation and Organizational Justice

Another variable we investigated regarding the predictor–outcome relationship articulated in Figure 1 is intrinsic work motivation. In general, work motivation is defined as the psychological force that generates complex cycles of goal-directed thought and behavior (Tziner et al., 2012). Motivation is what animates individuals to persist in courses of action until the acts are completed (Pinder, 2014). Accordingly, scholars studying work motivation attempt to articulate the processes by which an individual’s internal, psychological forces – in conjunction with external, environmental forces – determine the direction, intensity, and persistence of personal behavior aimed at goal attainment (Kanfer et al., 2017). Pinder (2014, p. 11) provides an alternative definition of work motivation as “a set of energetic forces that originate within individuals, as well as beyond an individual’s being, to initiate work-related behavior and to determine its form, direction, intensity, and duration.”

Intrinsic motivation occurs when individuals engage in goal striving because goal-pursuit activities are psychologically rewarding in themselves without links to external rewards (Ryan and Deci, 2017). Because those goal-pursuit activities are centered in the organizational environment, it follows that this type of work motivation (as well as other extrinsic types of motivation) results from the interaction between an individual’s characteristics and the external environment (Latham and Pinder, 2005), which we illustrate in the components of Figure 1. Research indicates that although compared to the intrinsic nature of goal-striving tasks and rewards in the external environment, individual difference characteristics may carry less weight in determining motivation, they are still critical components of determining the worth of outcomes because they are always active in determining motivation (Klein and Fein, 2005; Fein and Klein, 2011).

Intrinsic and extrinsic motivation are also key factors strongly related to goal content (Ryan and Deci, 2017). Goal content may reflect some mixture of intrinsic content, where behavior is pursued for the sake of engagement with an activity itself, and extrinsic content, which signals goals are pursued for the sake of external rewards. The literature strongly supports that intrinsic and extrinsic elements of goal content are differentially related to well-being outcomes, with intrinsic goals as antecedents of well-being (Dittmar et al., 2014).

Organizational justice is a state-based perception defined as the extent to which employees think or feel they are provided with appropriate, fair, and respectful treatment, adequate and accurate information, and reasonable resources and rewards (Cohen-Charash and Spector, 2001; Colquitt et al., 2001). These perceptions are a product of organizational occurrences and systems, often based on specific “organizational components,” such as leaders and co-workers (Hollensbe et al., 2008). Typically, organizational justice as an overall construct can be broken down into three facets, namely, *distributive justice* (fairness associated with decision outcomes and distribution of resources*), procedural justice* (fairness of the processes leading to outcomes), and *interactional justice* (the treatment an individual receives as decisions are made) (for further reading, see Niehoff and Moorman, 1993; Cohen-Charash and Spector, 2001; Colquitt et al., 2001). In the present study, we incorporated all three components to account for the full range of the justice construct.

Equity theory is the most important organizing principle surrounding the justice–motivation relationship (Adams, 1965). Namely, equity theory supposes that if employees experience some type of imbalance between their personal, perceived inputs into the workplace system, relative to their benefits received from the work system and structures, they will experience an adverse emotional state (due to perceived injustice, in this instance), and they will likely aspire to regain and maintain balance through some form of corrective behavior (Adams, 1965). In the case of experiencing organizational injustice, employees have been found to reduce their motivation and performance (Cohen-Charash and Spector, 2001; Viswesvaran and Ones, 2002). Moreover, the tenets of social exchange theory (SET) logic (Blau, 1986; see also Cole et al., 2002), suggest that lowering one’s motivation would appear to be a reasonable measure by which to address such imbalance.

It is clear that all sub-types of justice show positive associations with OCB, whether targeted to the organization or supervisor (Colquitt et al., 2013). This same meta-analytic evidence suggests that all sub-types of justice are negatively related to CWB, and in general the focus of CWB on organization or supervisor does not result in a difference in effect size (Colquitt et al., 2013). Trust, which is a crucial element within positive LMX and overall LMX quality, is also associated with higher levels of justice across all sub-types of justice (Colquitt et al., 2013). Thus, trust and LMX appear as key moderators of the justice-OCB relationship. Based on the integrative model presented in Figure 1, we expect intrinsic motivation and organizational justice to be inversely related to CWB.

### Counterproductive Work Behavior (CWB)

In recent years, CWB has gained much research attention because it has been shown to have important economic, sociological, and psychological implications in the workplace, including associations with unethical leader behavior (Bodankin and Tziner, 2009; Ho, 2012; Nei et al., 2018). Such dysfunctional behaviors include theft, sabotage, withdrawal, and harassment, among others (Bennett and Robinson, 2000; Spector et al., 2006). CWB almost invariably violates important organizational norms and harms organizations in ways associated with the organization’s goals, employees, procedures, productivity, and profitability (Spector et al., 2006). These behaviors may be directed against the organization itself or against its members, workers, and management alike, and hence they are costly to both individuals and organizations (Bennett and Robinson, 2000). Specific types of CWB include acting on negative feelings toward the organization via decreased motivation; manifesting distrust (toward the workplace and/or the managers); and even acting against the organization (Skarlicki and Folger, 1997). It makes sense that dysfunctional attitudes and behaviors of this nature are indicants of lack of job satisfaction, thus leading researchers in the field to hypothesize that work misbehavior is negatively associated with job satisfaction (e.g., Malhotra and Kathuria, 2017).

While most research has been conducted within the framework of goal content that benefits organizations, it is also appropriate to envision a type of negative goal content relative to organizational interests. Because CWB is considered to be an intentional behavior that is detrimental to organization interests, it could reflect a type of goal construct related to employee attempts to change their affective state within an organization (Dalal, 2005), which accords with evidence linking aggressive behaviors to attempts to change affective states (Bushman et al., 2001; Spector and Fox, 2002). This is consistent with several observations that the desire to change or maintain emotional states may serve as a common antecedent to both CWB and OCB (Spector and Fox, 2002; Dalal, 2005).

Furthermore, the relationship between CWB and organizational justice has been demonstrated, but that relationship seems to be contingent on other variables (Chernyak-Hai and Tziner, 2014). This is consistent with the notion that if employees experience aversion and imbalance (due to perceived injustice, in this instance), they will likely aspire to regain and maintain balance (Adams, 1965). Moreover, following the logic of SET, work misbehaviors would appear to be a reasonable measure by which to achieve that balance (Blau, 1986).

We also note that constructs that are antecedents to CWB may also serve as antecedents to Organizational Citizenship Behavior (OCB). In fact, the antecedents for OCB and CWB are very similar and should be related in opposing directions to job satisfaction, commitment, and justice (Dalal, 2005). Finally, concerning EI and its concordance with emotional regulation, and consistency with the mood regulation literature, both OCB and CWB can be considered adaptions – whereby the adaptive behaviors are meant to provide enhanced mood or satisfaction in the future (Dalal, 2005). Thus, such adaptions inherent in OCB and CWB may be geared toward the same goal of changing affect.

To increase our understanding of the nomological network around profiles of EI, work motivation, and justice, in the present study, we model CWB within the goal content component of Figure 1. Base on contingent relationships between CWB and other variables, as illustrated in Figure 1, we also decided to include three other critical constructs, described in the organizational psychology literature, that have been strongly associated with EI, namely, work motivation, justice, and CWB. These constructs are (a) *leader-member exchange* (LMX), which has been linked to CWB (Chernyak-Hai and Tziner, 2014); (b) *job satisfaction*, which has been associated with perceptions of organizational justice (Pignata et al., 2016); and (c) *burnout* (via emotional exhaustion), which is associated with a negative impact on employees’ attitudes toward work and work performance (Maslach et al., 2001) as well as rates of employee turnover (Wright and Cropanzano, 1998; Urien Angulo and Osca, 2012).

### Leader–Member Exchange (LMX)

The underlying proposition underlying LMX theory is that managers tend to employ different management styles for each of their subordinates (Graen and Uhl-Bien, 1995; see also Waismel-Manor et al., 2010). In turn, each specific relationship and corresponding management style induces corresponding differential responses and attitudes in subordinates, including different types of engagement (Aggarwal et al., 2020) and performance behaviors (Ilies et al., 2007). Capitalizing on reciprocity theory (Gouldner, 1960), employees in good or bad relationships with their managers (i.e., with high or low LMX) will feel obliged or reluctant to reciprocate these respective relationships (see also Adams, 1965).

Thus, high- or low-quality LMX results in correspondingly high or low levels of mutual trust, respect, and commitment. Accordingly, subordinates with high LMX relations are likely to receive more rewards (both formal and informal) than their colleagues with lower LMX relations. These benefits include tangible resources, career opportunities, emotional support (including emotional encouragement), and enhanced feedback (Graen and Uhl-Bien, 1995; Zagenczyk et al., 2015). Consequently, high LMX employees are more likely to engage in positive behaviors, including forgiving supervisor errors (Radulovic et al., 2019), while those low on LMX will be more prone to negative behaviors (Tziner et al., 2010; Breevaart et al., 2015). Conversely, and with respect to enlarging the network of constructs investigated in this study, it is important to note that poor relations between managers and their employees will almost certainly result in reciprocal counterproductive behavior (Chernyak-Hai and Tziner, 2014).

While LMX’s role as a potential mediator of workplace misbehaviors has been investigated (e.g., He et al., 2017), most studies emphasize contextual-level or job-level predictors (e.g., He et al., 2017; Sharif and Scandura, 2017). However, less is known about the effects of individuals’ dispositional differences on LMX (e.g., Maslyn et al., 2017; Hao et al., 2019). In addition, there is even less emphasis on the effects of cultural and demographic parameters on leader–member interrelations (for further reading, see Rockstuhl et al., 2012; Zagenczyk et al., 2015), which makes LMX worth including as a key state in this study.

### Job Satisfaction

Job satisfaction is defined as the pleasurable or positive attitude resulting from the overall positive evaluation of one’s job or work experiences. Job satisfaction is related to the extent that an individual’s needs are met in the work setting (Tziner et al., 2012), and consequently, job satisfaction can be linked to intrinsic factors, deriving from internally mediated rewards related to the essence of the job, and can also be linked to factors extrinsic to the individual, resulting from externally mediated rewards, such as adequate and appropriate pay (Porter and Kramer, 2004). For the purpose of enlarging the number of constructs investigated in this study, we note that job satisfaction is also associated with state-based perceptions of organizational justice (Tziner et al., 2011; Pignata et al., 2016). In addition, job satisfaction has also been shown to be related to individual characteristics, such as personal traits or dispositions (Tziner et al., 2008).

Meta-analytic evidence also suggests that job satisfaction and motivation are mediators that serve to enhance the relationship between LMX and performance (Martin et al., 2016) and that high LMX reduces the incidences of CWB. These findings suggest that the damaging effects of low LMX may more seriously affect performance through CWBs than previous research indicates (Martin et al., 2016).

### Burnout and Emotional Exhaustion

As opposed to job satisfaction, burnout is a progressive psychological response to chronic work stress that can be construed as a multidimensional construct involving three distinct but interrelated aspects, namely: (a) emotional exhaustion, (b) depersonalization (negative or cynical attitudes and feelings toward the organization and service recipients), and (c) a decline in personal accomplishment and in the perceived ability to perform effectively (Maslach, 2003). Notably, Shirom and Melamed (2006) also added physical fatigue to these dimensions of burnout.

Burnout has negative implications for employees’ state of health. For example, burnout is related to depression (Toker and Biron, 2012) and has also been found to be related to the increased risk of hyperlipidemia (Shirom et al., 2013), type-2 diabetes (Melamed et al., 2006), and inflamed levels of biomarkers such as C-reactive protein (Toker et al., 2005). Burnout is also an important component of general health outcomes that are related to total work hours and work–life conflict (Fein and Skinner, 2015).

This evidence suggests that burnout has clear implications for organizations, taking into account its negative impact on employees’ attitudes toward work and their work performance (Maslach et al., 2001). As burnout intensifies, it tends to induce lower levels of work satisfaction, which, in turn, enhance the rates of employee turnover (Wright and Cropanzano, 1998; Urien Angulo and Osca, 2012). Burned out employees may also influence colleagues negatively (Bakker et al., 2005) and burned out managers may exhaust the entire system they manage (Pines and Aronson, 1988). In the current study, of the three dimensions comprising burnout, we opted to survey only emotional exhaustion because, as reported in two recent meta-analyses, emotional exhaustion emerged as the most closely related to antecedents and outcomes of burnout (Lee et al., 2011; Cieslak et al., 2014). In addition, these three items of emotional exhaustion provided a uniform focus and maximum clarity of wording when measuring burnout.

Within intrinsic motivation frameworks, burnout can also be related to the failure to achieve goals based on corresponding failures of social exchange and affect regulation processes (Vansteenkiste and Ryan, 2013). These observations can be linked to the work of Colquitt et al. (2013), where the authors suggest numerous opportunities for integrating justice, CWB, and OCB through the integration of social exchange and affect processes as mediators. Although more work needs to be done regarding the relationship between social exchange and affect processes, Rupp et al. (2014) provide one explanation that includes social exchange as an amplifying mechanism that enhances the role of organizational justice. Based on these findings and the comprehensive model illustrated in Figure 1, we expect job satisfaction to be positively related to intrinsic motivation and emotional exhaustion to be negatively related to intrinsic motivation.

Within the present research, the framework we present in Figure 1 allows us to accomplish several important objectives:

• First, we can anchor the constructs of CWB and intrinsic motivation as central endogenous motivational processes.
• Second, in line with our reasons for conducting the present study, we were able to follow a model connecting key personal-level antecedents and reactions derived from the work context to the central endogenous motivational processes at the heart of our study, as well as to important work outcomes. As noted, the core purpose of our use of the Van den Broeck et al. (2019) model was the integration of the endogenous motivational processes surrounding CWB and intrinsic motivation with one critical personal level antecedent (EI) and two critical contextual-level antecedents (justice and LMX), which have been linked across numerous meta-analyses and previous studies (Dalal, 2005; Colquitt et al., 2013; Martin et al., 2016; Van den Broeck et al., 2016).
• Third, the use of the model afforded a logical link from antecedents (EI, justice, and LMX) and motivational processes (CWB and intrinsic motivation) to work outcomes, namely, job satisfaction (positive) and emotional exhaustion (negative), links which have been supported by previous studies (Vansteenkiste and Ryan, 2013; Martin et al., 2016; Miao et al., 2017).

Thus, the use of the Van den Broeck et al. (2019) model allowed us to broaden the scope of the current study to include antecedent constructs (EI, LMX, justice) endogenous motivational constructs (CWB, intrinsic motivation) and outcome-based constructs (job satisfaction and emotional exhaustion) in the same PAMS study. We note that because the PAMS approach is well suited to collective capturing and bringing together the impact of a relatively broad range of related variables, it was an appropriate method to use in testing the relationships with the framework displayed in Figure 1. Based on these associations, we outline how individuals’ perceptions of organizational justice serve as antecedents to motivation, as illustrated in Figure 1.

## Materials and Methods

### Participants

In the present study, we collected data from 3,293 Romanian participants, all employees from various telecommunications organizations (including high-tech, communications, and telemarketing, among others). The field research was based on the administration of questionnaires by students who participated as research assistants. The participation of the respondents in the survey was voluntary. In the questionnaire, the participants were assured of our respect for the principle of data confidentiality throughout the entire collection, processing, storage, dissemination, and archiving flow. Data regarding gender, age, professional experience, education level, and the exercise of a management activity were aggregated. Thus, the data become anonymous, making it impossible to identify the respondents. There are no questions in the questionnaire regarding the names, e-mail addresses, telephone numbers or other personal data of the respondents. In this way, the information was treated responsibly, according to European Union legislation in the field of personal data.

To minimize any potential nested effects of differences in organizational culture, because the Romanian corporate culture within telecommunications firms is relatively uniform, we focused exclusively on four telecommunications companies representative of the telecommunications industry. These included Vodafone, Orange, RCS&RDS, and Telekom. Table 1 incorporates the demographic information for these participants.

**TABLE 1:** Demographic information of study participants.

**Parameter**	**Category**	**Sample 1 (*n* = 3,293)**
		**(%)**
Gender	Males	60.00
	Females	40.00
Age	18–25	53.60
	26–35	23.20
	36–45	12.30
	46+	10.90
Education	High-school	31.2
	Tertiary	7.70
	Student/B.A. graduate	41.40
	Student/M.A. graduate and above	19.70
Tenure	0–5	66.10
	5–10	14.50
	10–15	7.50
	15–20	4.60
	20–25	2.80
	25+	4.40
Team work^a^	No	83.40
	Yes	16.60
Responsibility^b^	No	74.20
	Unit/team manager	15.70
	Department manager	6.80
	Director	3.40

*^*a*^Working in a team. ^*b*^Responsibility for other people’s work.*

### Procedure

The questionnaire was translated into Romanian by the fourth author of this paper, who is associated with the Bucharest University of Economic Studies, and Romanian is his maternal, education, and work language. The first author, who has equally mastered both the Romanian and English languages, compared the translations into Romanian against original English versions and essentially back-translated items from English to Romanian. Amendments to items were made if needed to ensure semantic equivalence. Only then was the questionnaire administered to participants. These instruments have already been in use in previous investigations in Romania.

A pencil and paper survey was given to working people in four telecommunications companies representative of the telecommunications industry, Vodafone, Orange, RCS&RDS, and Telekom, to complete voluntarily. After we collected the data, it was analyzed using the SPSS (v. 22.0) and AMOS (v. 22.0) software packages to assess multivariate normality. Consistent with the very large sample size, all variables were normally distributed. We considered the issue of common method variance (CMV) during the design of the study, and we used a number of design modifications to lower the risk of CMV. Although it was impossible for us to obtain data external to the questionnaire, we were able to position items measuring the CWB outcome further away from items assessing EI, motivation, and justice. We also reduced the emotional exhaustion items to the three items most clearly assessing burnout. Both of these adjustments are effective procedural remedies for CMV (Podsakoff et al., 2003).

### Measures

*Emotional intelligence* (EI) was measured using the Trait Emotional Intelligence Questionnaire-Short Form (TEIQue-SF; Petrides and Furnham, 2003), which includes 30 Likert-type items between 1 (very little) and 6 (very much); for instance, “I’m usually able to find ways to control my emotions when I want to.” Half the items were reverse-scored. In previous studies, the reliability coefficient (Cronbach’s α) of the questionnaire ranged between 0.82 and 0.89 (Pérez et al., 2005; Petrides and Furnham, 2006; Cooper and Petrides, 2010). In the current study, the measure had strong reliability: α = 0.91 (*M* = 4.26; *SD* = 0.96).

*Work motivation* (MO) was gauged by the Work Extrinsic and Intrinsic Motivation Scale (WEIMS; Tremblay et al., 2009), consisting of 18 Likert-type items ranging from 1 (does not correspond at all) to 6 (corresponds exactly); for example, “The reason for being involved in my job is for the satisfaction I experience when I am successful at doing difficult tasks.” In the present study, we used the intrinsic dimension of the scale. The measure had high reliability: α = 0.92 (*M* = 4.12; *SD* = 0.87).

*Organizational justice* (OJ) was measured using the Justice Scale (Niehoff and Moorman, 1993), which includes 20 Likert-type items between 1 (completely disagree) and 6 (completely agree); for instance, “I consider my workload to be quite fair.” The mean reliability coefficient of the questionnaire was 0.84 (Niehoff and Moorman, 1993). In the current study, the measure had strong reliability: α = 0.96 (*M* = 4.13; *SD* = 0.96). The three subscales of this construct were measured as follows: *organizational justice-distributive* (DI) comprised five items (α = 0.83; *M* = 4.11; *SD* = 1.07); *organizational justice-procedural* (FP) consisted of six items (α = 0.88; *M* = 4.10; *SD* = 1.03), and *organizational justice-interactive* (IJ) was gauged by nine items (α = 0.89; *M* = 4.19; *SD* = 1.02).

*CWB* was measured by employing the Interpersonal Deviance (ID) and Organizational Deviance (OD) Scale (IODS; Bennett and Robinson, 2000), which includes 18 Likert-type items between 1 (never) and 6 (every day); for instance, “I deliberately worked slower than I could.” The mean reliability coefficient of the questionnaire was 0.80 (Bennett and Robinson, 2000). In the current study, the measure had strong reliability: α = 0.96 (*M* = 1.98; *SD* = 1.03). Moreover, the reliability for *interpersonal deviance* (ID) was 0.87 (six items, *M* = 1.98; *SD* = 1.08) and the reliability for *organizational deviance* (OD) was 0.94 (12 items, *M* = 2.0; *SD* = 1.05).

*LMX* was gauged by the LMX7 questionnaire (LMX7; Graen and Uhl-Bien, 1995), consisting of seven Likert-type items; however, each item had a different scale, from 1 (rarely, not a bit, not at all, none, strongly disagree, extremely ineffective) to 6 (very often, a great deal, fully, very high, strongly agree, extremely effective). Original reliability was α = 0.91. In the current research, reliability was: α = 0.86 (*M* = 4.11; *SD* = 0.91).

*Job satisfaction* (SA) was tapped with the MSQ 20-item questionnaire (Weiss et al., 1967). Each item of the questionnaire assesses a facet of work satisfaction; for instance, “To what extent are you satisfied with the chance to do something that makes use of your abilities?” The responses were given on a six-point scale. In a previous study (Smith and Tziner, 1998), the reliability coefficient of this measure was 0.82. The reliability in this paper was: α = 0.96 (*M* = 4.35; *SD* = 0.88).

*Emotional exhaustion* was measured using the Maslach Burnout Inventory (MBI; Maslach et al., 1986). As indicated, of the three dimensions of burnout, in this paper, we used only emotional exhaustion (EE), comprising nine Likert-type items between 1 (a few times a year) and six (every day); for instance, “I feel emotionally drained from my work.” In a previous study (Smith and Tziner, 1998), Cronbach’s α of this measure was 0.89. In the current study, the measure had strong reliability: α = 0.92 (*M* = 2.76; *SD* = 1.06).

### Control Variables

Past empirical research has found no evidence of a meaningful relationship between demographic characteristics and research variables. In this study, all correlations between the demographic variables (age, education, tenure, teamwork, and responsibility) and the investigated variables were below 0.1; therefore, none of these variables were controlled in subsequent analyses.

Table 2 presents the validity indices for the measures used in the research, based on confirmatory factor analysis (CFA).

**TABLE 2:** Validity indices for the measures used in the research, based on CFA.

**Measure**	**CR**	**AVE**	**MaxR(H)**
Emotional exhaustion	0.92	0.53	0.92
CWB (interpersonal)	0.87	0.53	0.92
CWB (organizational)	0.94	0.58	0.95
EI	0.91	0.44^a^	0.93
Work motivation	0.92	0.41^a^	0.94
Job satisfaction	0.96	0.57	0.97
LMX	0.86	0.46^a^	0.87
Distributive justice	0.83	0.50	0.83
Procedural justice	0.88	0.55	0.89
Interactional justice	0.89	0.48^a^	0.92

*^*a*^Convergent validity issue as the average variance extracted (AVE) < 0.50. CR, composite reliability. MaxR(H), maximum reliability. CWB, counterproductive work behavior. EI, emotional intelligence. LMX, leader–member exchange.*

### PAMS Approach

In the social sciences, one of the most popular representations of data is a tabular form where rows represent cases (e.g., people) and columns represent measurements of variables (e.g., items or subscales). We can view rows – arrays of column subscale scores – as person profiles. Each person profile carries two types of information: (1) the summary statistics (quantitative) that represent the profile level or height, and (2) the contextual pattern that the profile exhibits (qualitative) in each individual’s person profile of observed score.

In the present study, using Profile Analysis via Multidimensional Scaling (PAMS; Kim et al., 2017; Kim and Kim, unpublished), we analyzed 3,293 cases or individuals who were measured by six subscales (EI = emotional intelligence; MO = work motivation; LMX = leader-member exchange; DI = organizational justice-distributive; FP = organizational justice-procedural; IJ = organizational justice-interactive) to capture both quantitative profile-level information and contextual profile pattern information (i.e., two core profile patterns identified in the present study). The profile level is the average of input variable scores. In the present study, the profile level was, in fact, the average of six subscale scores, and there were 3,293 profile levels. The profile pattern information appears in ipsatized scores around the person level.

**FIGURE 2:**
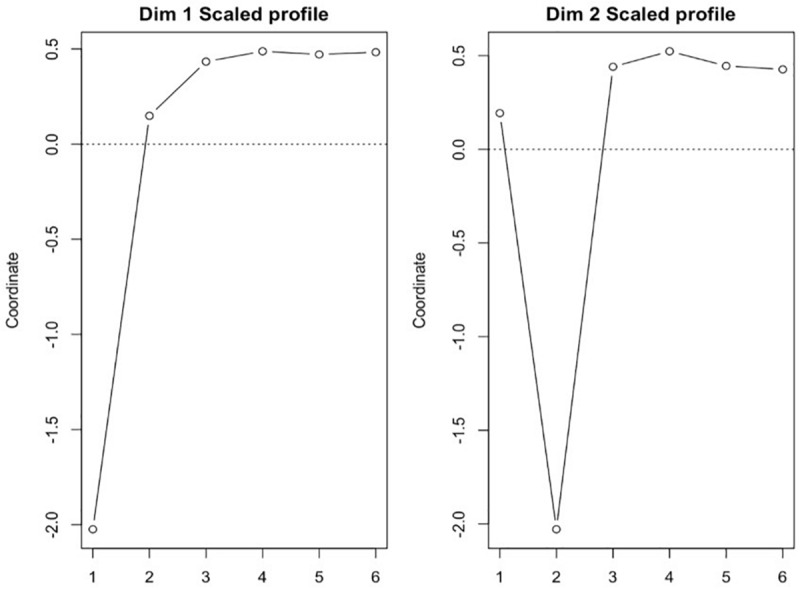
Patterns of dimensional profiles, where 1 = emotional intelligence; 2 = work motivation; 3 = leader-member exchange; 4 = organizational justice-distributive; 5 = organizational justice-procedural; 6 = organizational justice-interpersonal.

For example, we can assume person *p*’s level to be Cp (*p* = 1, …, 3,293 in the study) and each subscore to be Mpj (*j* = 1, …, 6) since there were six subscores used as input variables for PAMS in this study, and then an array of person p’s ipsatized subscores, (Mp1 – Cp), …, (Mp6 – Cp), represents the person *p*’s profile pattern. PAMS uses only this person pattern information to identify core profiles. In the present study, PAMS analyzed 3,293 arrays of six ipsatized subscores to identify two core profiles (see Figure 2) (see Kim, 2013; Kim and Kim, 2017, for details).

Notably, the PAMS approach can be interpreted as a type of dimensionality reduction technique for person profiles. Thus, regarding the option to choose between person-centering and group-centering (organization-level centering in our case), estimation of core profiles in the PAMS model is based on a *person-centering*, but not on a group-centering because PAMS estimates within-person variation in a given population. Therefore, organization-level centering is not considered in the PAMS model.

PAMS attempts to identify the most typical response patterns (called dimensional profiles) in a population and then summarizes individuals as linear combinations of these dimensional profiles. PAMS identifies two or three of the dimensional profiles that represent the most typical response profiles in a dataset. One can thus describe each individual’s profile as the linear combination of these dimensional profiles. This representation is parameterized by regression coefficients (known as person weights), one for each dimensional profile. Notably, person weights are useful because they relate individual profiles to core profiles in an interpretable way.

Furthermore, although PAMS is a multivariate statistical method, unlike traditional methods, it does not require the assumption of normality, is easy to use, and effects can be detected in smaller samples (even sample sizes of fifty or less). We applied the PAMS paradigm to the Romanian sample data to identify dimensional profiles regarding organizational justice perceptions and personal states. In Table 3, a few person parameter estimates are included by way of example.

**TABLE 3:** Example data for interpretation of person weights.

**id**	**w1**	**w2**	**level**	**R^∧^2**	**corDim1**	**corDim2**
#1	0.83	−1.78	3.28	0.47	0.57	−0.56
#3	1.88	−0.19	4.46	1.00	1.00	−0.72
#32	−0.47	1.78	3.85	0.89	−0.83	0.93

*Where w1 = person regression weight_1; w2 = person regression weight_2; level = person average score for six subscales included as input variables in PAMS; R^∧^2 = R-squared (which is a proportion of person variance occurring in his/her profile accounted for by two core profiles); corDim1 = partial correlation of a person between a person profile and core profile_1; corDim2 = partial correlation of a person between a person profile and core profile.*

Regarding Table 3, if someone (e.g., #3) has a high correlation with core profile 1 (based on the partial correlation for corDim1 x #3), that person’s profile pattern would be similar to the core profile 1 pattern. Thus, the data from Table 3 shows that the profile of #3 is essentially identical to the core profile 1. On the other hand, if someone has a high correlation with core profile 2, it is expected that that person’s profile would be similar to core profile 2. In the case of the data in Table 3, this relationship is revealed for #32. For details, please consult the previous PAMS studies (Kim et al., 2004, 2007, 2017; Frisby and Kim, 2008; Kim and Kim, 2017).

We chose PAMS instead of other methods of multivariate data analysis because we wanted to identify the central response patterns (the so-called “core” profiles in the present study) in a given sample. The core profiles are similar to factors extracted in exploratory factor analysis in terms of a dimension-reduction technique. However, the PAMS approach was better suited to this project because researchers can extract a few core profiles (two or three) out of numerous person profiles of observed scores in a sample. Note that PAMS views cases (or rows) in a dataset as arrays of observed column variable scores, which are called “person profiles” in PAMS terms.

However, there are fundamental differences between PAMS and other dimension-reduction methods, especially factor analysis (FA) (either exploratory or confirmatory). First, extracted factors represent certain latent traits included in a sample (e.g., intelligence or personality); PAMS, however, does not “seek” latent traits in a given sample.

Second, in the present study, PAMS identifies and extracts two core profiles from a dataset compiled from a sample of 3,293 person profiles of six observed scale scores (EI, MO, LMX, DI, FP, and IJ). Peaks on certain subscales represent high scores (because of personal skills, inclinations, or preferences on the subscale measurements) and valleys represent low scores (because of lack of skills or inappropriateness on the subscale measurements) (For details, see Kim et al., 2004, 2007; Kim, 2013).

Third, unlike factors or FA results, PAMS is designed to replicate person profiles that incorporate core profiles, based on the assumption that (a) person profiles are linearly related to core profiles as in multiple regression, and (b) that person profiles are considered as response variables (as in regression), and core profiles as predictor variables.

Finally, FA “attempts” to group homogeneous variables (by a rotation method) in several clusters and interprets these clustered variables as factors, pursuing “a simple structure” where a variable is assumed to be loaded onto one factor to enhance interpretation of factors, and factor loadings are considered to be unidirectional (usually positive). However, in PAMS, the directions of core profile coordinates (analogous to factor loadings) are irrelevant; and no rotation is required to enhance interpretation because all the input variables (six subscales in our study) are used to characterize each core profile pattern. For these reasons, rather than employ multivariate analytical methods, such as factor analysis, SEM, or HLM, we used PAMS as our primary analysis tool.

In addition, convergent and discriminant validity is addressed via the correlations between two core profiles and a third variable such as emotional exhaustion (EE), interpersonal deviance (ID), organizational deviance (OD), and job satisfaction (SA). For convergent validity we can use the examples of r(EE, dimension 1) = –0.56^∗∗^ and r(EE, dimension 2) = –0.060^∗∗^ with r(SA, dimension 1) = 0.65^∗∗^ and r(SA, dimension 2) = 0.07^∗∗^ as reflected in Table 2. Convergent validity is indicated because correlation coefficients for dimensions 1 and 2, for both EE and SA, the same directions were indicated, although the magnitudes were different. Within PAMS this indicates evidence of convergent validity for the dimension profiles. Conversely, for discriminant validity we use the example of r(ID, dimension 1) = –0.43^∗∗^ and r(ID, dimension 2) = 0.10^∗^ with r(OD, dimension 1) = –0.44^∗∗^ and r(OD, dimension 2) = 0.13^∗∗^. Here the different directions of correlation coefficients between the two core profiles within both ID and OD are evidences of discriminant validity for the dimension profiles. Also, the correlation between the dimension profiles was r(dimension 1, dimension 2) = 0.12^∗∗^. Because of a extremely reduced standard error caused by a large sample size (*N* = 3,293), this small correlation coefficient was statistically significant at *a* = 0.01, but the squared of 0.124 equals to 0.015 means that about 1.5% variance was shared between two core profiles (extracted from the present data set). This correlational result implies discriminant validity for the core profiles extracted from the current data set.

## Results

Next, to describe the network of associations among the study’s variables, a Pearson correlation matrix was derived, as presented in Table 4.

**TABLE 4:** Correlation matrix (*n* = **3,293**).

	***M***	***SD***	**1**	**2**	**3**	**4**	**5**	**6**	**7**	**8**	**9**	**10**	**11**	**12**	**13**	**14**	**15**	**16**
(1) Gender			1	0.06**	–0.02	0.06**	–0.01	0.12**	–0.03	−0.05**	0.01	−0.08**	−0.08**	−0.06**	–0.03	0.15**	0.14**	–0.01
(2) Age				1	0.13**	0.77**	0.10**	0.22**	−0.07**	0.06**	−0.05**	–0.02	0.01	–0.01	0.00	−0.06**	−0.06**	−0.05**
(3) Edu					1	0.09**	–0.01	0.20**	−0.09**	0.06**	0.05**	0.02	0.03	0.03	–0.02	−0.05**	−0.05**	0.05**
(4) Tenure						1	0.08**	0.27**	−0.04**	0.04**	–0.02	–0.02	0.01	–0.01	0.00	–0.02	–0.01	−0.04*
(5) Teamwork							1	−0.04*	0.04*	−0.03*	−0.08**	−0.08**	−0.07**	−0.06**	0.07**	–0.01	0.03	−0.12**
(6) Managerial level								1	–0.03	0.05**	0.07**	0.01	0.03*	0.03	–0.01	0.11**	0.10**	0.08**
(7) Emotional intelligence	4.26	0.96							1	−0.07**	−0.19**	−0.23**	−0.24**	−0.22**	0.53**	0.43**	0.47**	0.06**
(8) Work motivation	4.12	0.87								1	0.30**	0.52**	0.55**	0.53**	−0.14**	−0.10**	−0.17**	0.27**
(9) LMX	4.11	0.91									1	0.54**	0.53**	0.58**	−0.27**	−0.07**	−0.13**	0.33**
(10) Org. justice -distributive	4.11	1.07										1	0.84**	0.86**	−0.28**	−0.23**	−0.28**	0.34**
(11) Org. justice -procedural	4.10	1.03											1	0.88**	−0.28**	−0.23**	−0.27**	0.32**
(12) Org. justice -interactive	4.19	1.02												1	−0.29**	−0.18**	−0.23**	0.35**
(13) Emotional exhaustion	2.76	1.06													1	0.28**	0.31**	0.04**
(14) CWB-I	1.98	1.08														1	0.84**	0.17**
(15) CWB-O	2.00	1.05															1	0.12**
(16) Job satisfaction	4.35	0.88																1

*p < 0.05, **p < 0.01. (1) Pearson correlations are shown above. Frequencies for Gender, Age, Education, Tenure, Teamwork and Managerial Level appear in Table 1.

Utilizing the enhanced PAMS (Kim et al., 2017; Kim and Kim, unpublished), we identified two *two*-*dimensional profiles* from the six predictor variables included in the current data, namely: emotional intelligence (EI); work motivation (MO); leader–member-exchange (LMX); organizational justice-distributive (DI); organizational justice-procedural (FP); and organizational justice-interactive (IJ).

We identified two core profiles from the present dataset based on two criteria: stress and interpretability. Stress is analogous to Steiger’s RMSEA (root mean square error of approximation) or inverse of TLI (Tucker–Lewis Index) in SEM, and a value of 0.05 or less of stress signifies goodness-of-fit (to data) for proposed dimensionality. The stress value of the proposed two-dimensional solution in our study was 0.0022, and its bootstrap empirical confidence interval was (0.0011, 0.0058), verifying the stability of the two-dimensional solution. From 3,293 person profiles of the six observed measurements, using PAMS, we identified two core profiles that accounted for 68% of variance occurring in 3,293 person profiles.

Also, this two-dimensional solution satisfied the interpretability of the dimensions based on our judgment from the standpoint of organizational psychology.

The person weights are in fact regression weights/coefficients estimated by regressing the person profiles of the six observed measurements (EI, MO, LMX, DI, FP, and IJ) included in our data matrix that consisted of 3,293 cases (rows) and six organizational measures loaded on to two core profiles. The “person” weights function as matching statistics between person profiles and core profiles in terms of their patterns and they are often expressed in terms of the correlations between them (Kim and Kim, 2017): Each person (of the 3,293) is assigned two “person” weights in our study because we identified two core profiles.

With the coordinates included in Table 4, we generated patterns of the dimensional profiles (see Figure 2). According to the profile pattern information generated, we labeled the dimension 1 profile as *high overall justice and low emotional intelligence* and the dimension 2 profile as *high emotional intelligence and low work motivation*. With reference to the integrated framework in Figure 1, each of these profiles represents a different state or arrangement of variables with the framework. Specifically, for the dimension 1 profile, we assume that justice perceptions are fixed at a high level within this state and EI is fixed at a low level within this state, while all other measured elements are aligned with their high or low positions in Figure 2. Similarly, for the dimension 2 profile, we assume that EI is fixed at a high level within this state and that intrinsic motivation is fixed at a low level within this state, while all other measured elements are aligned with their high or low positions in Figure 2.

In reality, the patterns of the dimensional profile should represent the pattern of the means of the six predictor variables. For example, if one plotted the six predictor variables’ means in a spreadsheet file (i.e., a predictor variable-mean profile for the dimension 1 profile), its profile pattern should be equal to the pattern of the dimension 1 profile. To confirm this, we estimated the correlation between the predictors’ mean profile and the dimension 1 profile. The correlation was 0.99, indeed indicating that the two patterns were visually identical.

### Validation of Core Profile Patterns

To validate core profile patterns identified from the sample (*n* = 3,213), we first randomly split our original sample into two: Sample 1 as a calibration sample (*n* = 1,606) and Sample 2 as a validation sample (*n* = 1,607), and we then compared the two core profile patterns from Samples 1 and 2. The correlation between the first core profiles from Samples 1 and 2 was 1.00, and the correlation between the second core profiles from Samples 1 and 2 was 0.99. As expected, the correlations between the core profiles of the whole sample and the core profiles of the validation sample (Sample 2) were between 0.99 and 1.00.

**FIGURE 3:**
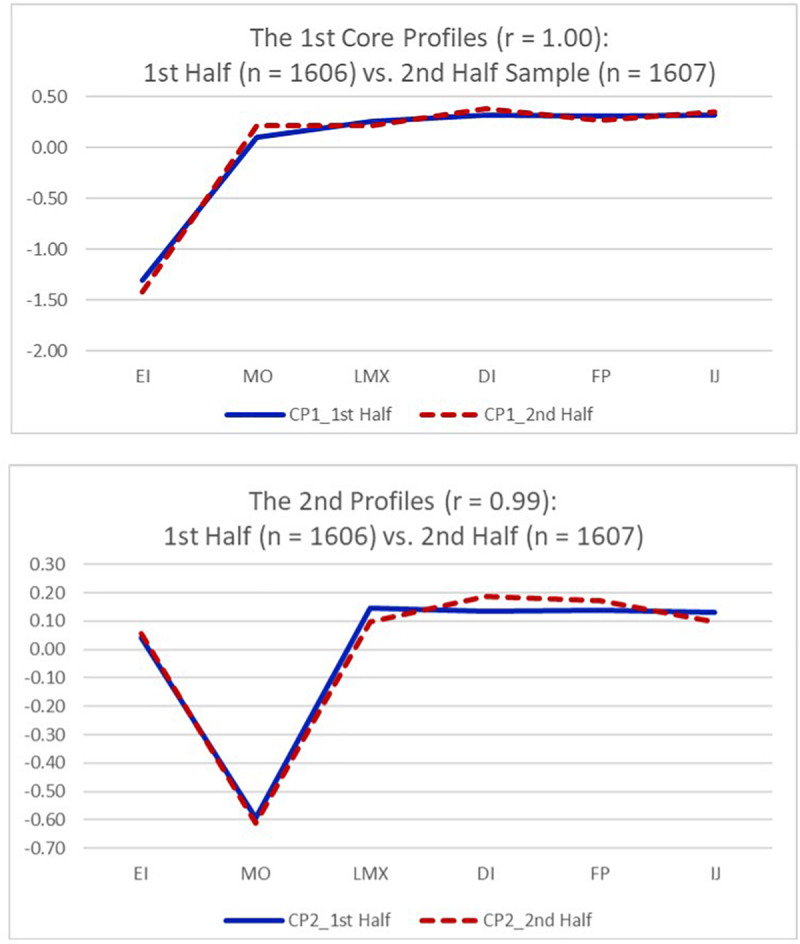
Comparing the core profile patterns in the first and second half samples.

We included both profile coordinates in a table (Table 5) and juxtaposed profile patterns in a figure (Figure 3). Since there was no difference in core profile patterns between the calibration sample (Sample 1) and the validation sample (Sample 2), we kept our original profiles estimated from the whole sample because they were almost identical to those core profiles estimated from Sample 1 (for calibration) and Sample 2 (for validation). Table 5 provides the core profile coordinates from the calibration and validation samples and for the whole sample. Figure 3 illustrates that the PAMS responses between the calibration and validation samples are visually identical. In sum, all core profile patterns identified from Sample 1, Sample 2, and the whole sample were virtually the same, and we thus included the core profiles of the entire sample as the final ones.

**TABLE 5:** The core profile coordinates from split halves and whole samples.

	**CP1_1st Half**	**CP1_2nd Half**	**CP1_Whole**
EI	–1.30	–1.42	–1.30
MO	0.10	0.21	0.10
LMX	0.25	0.22	0.28
DI	0.32	0.38	0.31
FP	0.31	0.26	0.30
IJ	0.32	0.35	0.31

	**CP2_1st Half**	**CP2_2nd Half**	**CP2_Whole**

EI	0.04	0.06	0.06
MO	–0.59	–0.61	–0.60
LMX	0.15	0.10	0.13
DI	0.14	0.19	0.15
FP	0.14	0.17	0.13
IJ	0.13	0.10	0.13

*CP, core profile; 1st Half, the first randomly split half sample (*n* = 1,606); 2nd Half, the second randomly split half sample (*n* = 1,607); Whole, the whole sample (*n* = 3,213). EI, emotional intelligence; MO, work motivation; LMX, leader–member exchange; DI, organizational justice-distributive; FP, organizational justice-procedural; IJ, organizational justice-interactive.*

The two profiles, incorporating the six predictor variables scores, accounted for 68% of the total variance occurring within the 3,293 individuals’ response profiles. The stress value for the two-dimensional (profiles) solution was 0.0022, indicating the goodness-of-fit. To test its statistical meaningfulness, we generated 2,000 bootstrap samples and estimated its 95% biased-corrected and accelerated (BCa) bootstrap confidence interval for the stress value. The confidence interval was (0.0011, 0.0058), which confirmed its statistical significance at α = 0.05.

Utilizing the 2,000 bootstrap samples, we also estimated the 95% BCa confidence intervals for the coordinates of the dimensional profiles. The coordinates whose confidence intervals included zeros were considered statistically insignificant. Table 6 consists of a summary of these results.

**TABLE 6:** Dimensional profile coordinates, mean coordinates, standard errors, and confidence intervals for the whole sample.

	**Original**	**Mean**	***SE***	**BCaLower**	**BCaUpper**
**Dimension 1**					
EI	−**1.30**	–1.33	0.06	–1.42	–1.23
MO	0.10	0.11	0.05	–0.02	0.22
LMX	**0.28**	0.21	0.06	0.22	0.38
DI	**0.31**	0.35	0.03	0.25	0.33
FP	**0.30**	0.32	0.04	0.19	0.36
IJ	**0.31**	0.34	0.02	0.29	0.31
**Dimension 2**					
EI	**0.06**	0.03	0.03	0.02	0.09
MO	−**0.60**	–0.43	0.21	–0.63	–0.24
LMX	0.13	0.07	0.11	–0.09	0.23
DI	0.15	0.16	0.08	–0.01	0.31
FP	0.13	0.10	0.08	–0.04	0.24
IJ	0.13	0.07	0.08	–0.02	0.24

*Significant coordinates are in bold. Original, indicates the original coordinates before bootstrapping. Mean, the mean coordinates from 2,000 bootstrap coordinate estimates. SE, bootstrap standard error estimates for the coordinates. BCaLower, the 2.5th percentile lower tail and BCaUpper, the 97.5th percentile upper tail. EI, emotional intelligence; MO, work motivation; LMX, leader–member exchange; DI, organizational justice-distributive; FP, organizational justice-procedural; and IJ, organizational justice-interactive.*

To examine the utility of the dimensional profile information across all observations, we estimated correlations between four of the dependent variables in the current study and the dimensional profiles. The dependent variables were emotional exhaustion (EE), interpersonal deviance (ID), organizational deviance (OD), and job satisfaction (SA). The correlations were:

$$r\,\left(\mathrm{EE},\mathrm{dimension}\,1\right)=-0.56^{**}\,\mathrm{and}$$

$$\left(\mathrm{EE},\mathrm{dimension}\,2\right)=-0.060^{**}$$

$$r\,\left(\mathrm{ID},\mathrm{dimension}\,1\right)=-0.43^{**}\,\mathrm{and}$$

$$r\,\left(\mathrm{ID},\mathrm{dimension}\,2\right)=0.10^{*}$$

$$r\,\left(\mathrm{OD},\mathrm{dimension}\,1\right)=-0.44^{**}\,\mathrm{and}$$

$$r\,\left(\mathrm{OD},\mathrm{dimension}\,2\right)=0.13^{**}$$

$$r\,\left(\mathrm{SA},\mathrm{dimension}\,1\right)=0.65^{**}\,\mathrm{and}$$

$$r\,\left(\mathrm{SA},\mathrm{dimension}\,2\right)=0.07^{**}$$

where ^∗∗^ refers to *p* < 0.01.

Thus, EE, ID, and OD variables had *negative* and statistically significant correlations with the dimension 1 profile, whereas SA had a *positive* and statistically significant correlation with the dimension 1 profile. EE had a *negative* and statistically significant correlation with the dimension 2 profile. However, ID, OD, and SA had *positive* and statistically weak but significant correlations with the dimension 2 profile. We note that these finding are in according with other studies testing relationships between EI, motivation, and job satisfaction (Carmeli, 2003; Christie et al., 2007; Othman et al., 2009).

In addition, for both the dimension 1 profile and the dimension 2 profile, the PAMS method allows users to envision an *inverse profile* for each dimension, in which each of the maker variables indicated as fixed in reference to the initial profile is then envisioned at the opposite ends of the scale for the inverse profile. For example, the substantial negative correlations with the dimension 1 profile indicate that those responders who scored high on EE, ID, and OD are *inversely* related to the dimension 1 profile pattern – the *high overall justice and low emotional intelligence*. Thus, concomitantly, those same participants, scoring high on EE, ID, and OD, will align with an inverse profile pattern (of the six predictor scores included in PAMS) that indicates an inversely related *low overall justice and high emotional intelligence* profile. In contrast, within the basic dimension 1 profile, those who have low EE, ID, and OD scores tend to have a *high* overall justice component score, but a *low* emotional intelligence score.

Additionally, respondents demonstrating high ID, OD, and SA exhibit weak, yet statistically significant positive, correlations with the *high emotional intelligence and low work motivation* profile (dimension 2). Thus, these respondents also tend to have their score response patterns resembling dimension 2, the *high emotional intelligence and low work motivation* profile. Conversely, employees experiencing high EE scores tend toward the *low emotional intelligence and high work motivation* profile (the inverse profile for dimension 2).

## Discussion

The use of the Van den Broeck et al. (2019) model allowed us to broaden the scope of the present study to include antecedent constructs (EI, LMX, justice), endogenous motivational constructs (CWB, intrinsic motivation), and outcome-based constructs (job satisfaction and emotional exhaustion) in the same PAMS study.

The first indication from this study is that rather than the individual components of justice (see above) impinging upon employees’ exhaustion and workplace deviance, it is the *overall* perception of justice that affects the deviant employees. This finding is similar to that of previous research on this topic (e.g., Chernyak-Hai and Tziner, 2014). Thus, based on the inverse profile for dimension 1 in this study, low, *overall* organizational justice and high emotional intelligence are commensurate with high emotional exhaustion, interpersonal deviance, and organizational deviance.

Concerning the consequences of this perceived sense of injustice, the finding is congruent with those emanating from previous studies, such as those of Maslach et al. (2001); Moliner et al. (2005), and Shkoler and Tziner (2017) that illustrate that a sense of unfairness could eventually lead to burnout, of which emotional exhaustion constitutes a major facet (Son et al., 2014). Indeed, as noted, injustice in the workplace consumes employees psychologically and depletes their personal resources over time (Hobfoll, 1989), thus inducing a state of emotional exhaustion (Tepper, 2001).

Conversely, our finding that a high perception of organizational justice and fair treatment links very positively with job satisfaction is not surprising; it coheres with both social exchange theory (Blau, 1986; see also Cole et al., 2002) and the extant literature (Pignata et al., 2016; Mashi, 2018).

The relationship between low organizational justice and high levels of emotional intelligence, recorded above, is seemingly unexpected, for we recall that high EI should essentially enhance the control of emotions and the handling of difficult situations in life (Joseph and Newman, 2010). A plausible explanation, however, for this found relationship between (overall) justice and workplace deviance among the high-EI subjects is that highly emotionally intelligent employees actually manipulate those frustrations founded upon perceived injustice in a sophisticated and malicious way (e.g., Shkoler and Tziner, 2017): they harm other employees and the organization as a whole. However, this subtle Machiavellian behavior depletes psychic-energetic resources because of the need to be constantly alert in order to keep this misbehavior hidden. This defensive posture, in turn, is likely to provoke emotional exhaustion.

Our results indicate that the opposite relationship of the above also holds true, namely, that those who demonstrate *high* levels of organizational justice, along with *low* levels of emotional intelligence, also tend toward higher levels of emotional exhaustion, interpersonal deviance, and organizational deviance. Possibly, from a psychological weighting perspective, lower EI, which is associated with lack of self-regulation and monitoring of emotions, would appear to have a more powerful impact on emotional exhaustion and organizational deviance than high justice perceptions.

Of interest, our study revealed a dimension 1 profile labeled *high overall justice and low emotional intelligence*, indicating that high job satisfaction is consistent with low emotional intelligence. At first glance, this might seem contradictory to the above-mentioned finding that this combination leads to higher levels of emotional exhaustion, interpersonal deviance, and organizational deviance. Indeed, a review of the literature indicates mixed results in this respect, with some studies reporting a positive relationship between job satisfaction and emotional intelligence (e.g., Rezvani et al., 2016), and others indicating a negative relationship (e.g., Thompson and Samuel, 2014).

While the former link is intuitively conceivable, the latter relationship is puzzling, as we expect highly emotional intelligent individuals to be inclined toward utilization of their cognitive capacity of coping with negative emotions that emanate from their jobs (and other work-related contextual factors) (Dilchert et al., 2007). Indeed, we anticipate that high-EI employees develop positive emotions at work, experience the job as pleasant and gratifying, and achieve feelings of high job satisfaction (Adil and Kamal, 2016).

Conversely, we expect individual employees characterized by *low* EI to be unlikely to cope with their negative feelings and prone to release their frustration through disobedience. Alternatively, these low-EI employees would be disposed to display “fake” emotions (i.e., surface acting), which entails suppressing negative feelings and a substantial investment of psychological energy (e.g., Prati et al., 2009), resulting in lower levels of job satisfaction.

Nevertheless, beyond these hypothetical contemplations, in our study we observe clearly that the relationship is inverse, namely, that low emotional intelligence is consistent with high job satisfaction. Perhaps, reverting back to our assessment of high-EI employees, we might now propose that low-EI employees demonstrate a lower capacity for complex schemes (such as the Machiavellian ploys described above). Moreover, from a psychological weighting perspective, the combined high perceptions of justice overcome any tendencies toward deviant behavior, such that the final existential state is one of high work satisfaction. Alternatively, we might contend, in contradistinction to the previous proposition, that low-EI employees would not, *a priori*, make efforts to “surface act.” More likely, they may innocently adopt a more naive or simple perspective of their job and, as such, more easily find work satisfaction.

We would like, additionally, to cast an interpretative light on the findings related to dimension 2, the *high emotional intelligence and low work motivation* profile that revealed an associate pattern score characterized by high interpersonal deviance, organizational deviance, and low job satisfaction. As discussed, it is easily understandable that when motivation at work is at a low level, employees are more prone to experience low levels of job satisfaction and to display work misbehavior (Shkoler and Tziner, 2017). We also observed that high-EI employees with low job motivation, based on perceived injustice, fathom out ways to harm other employees and the organization as a whole (Joseph and Newman, 2010). Thus, this employee profile must surely represent a “red light” for managers in the workplace.

Last, we might also need to inquire why the combination of low levels of emotional intelligence and high work motivation is associated with a high level of emotional exhaustion. One possible response is that high levels of work motivation compel employees to expend considerable energy at work, and that the burned-up energy amounts to exhaustion that constitutes a severe depletion of personal resources. Moreover, low emotional intelligence hampers effective assessment of feelings, such that the poor emotional regulation also induces and contributes toward a state of emotional exhaustion (Adil and Kamal, 2016).

## Conclusion

The major contributions of this research are found within (a) the conceptual unification of goal choice and goal-striving processes linked to state-based and individual difference antecedents, and (b) within the empirical support generated for this unified motivational framework in the development and demonstration of two distinct person profiles. We demonstrated the utility of the overarching motivational framework using PAMS across a total sample of 3,293 respondents. Moreover, we note the abundance of research supporting the necessity of a model to serve as an advanced organizer as part of guided instruction in complex environments (White, 1992; Mayer, 2004; Kirschner et al., 2006).

In light of these observations, we recommend to practitioners that train managers to understand the complexity of the related effects that impact on motivation in the workplace, that they use heuristic conceptual frameworks combining multiple motivational perspectives into a unified model similar to the type used within this paper. Such an approach allows for the unification of isolated motivational models, which must be understood together for managers to appreciate the complex interrelationships between goal choice, goal striving, and state and individual difference antecedents to motivation, and their ultimate connections to organizational outcomes. For example, there are several reasons why individuals choose to engage in CWB that include reactions to injustice or job dissatisfaction, negative role models, and thrill-seeking (Bennett and Robinson, 2000). The results of our research suggest that when choices to engage in CWB occur in contexts of perceived injustice, a high level of EI may actually be positively associated with CWB.

### Limitations and Future Research

We note that the paths illustrated in this research do not provide *direct* causal evidence. However, these paths do indicate very strong networks of association that might be leveraged to explain in more detail focal constructs such as emotional intelligence. In addition, our measurements were based on self-report questionnaires. Although we attempted to employ procedural remedies to minimize the risk of significant common method variance (CMV), multiple self-report questionnaires may contain some shared variability due to measurement methods, although any potential bias due to CMV is likely to be very low (Spector, 2006). However, following expert recommendations (Podsakoff et al., 2003) future researchers should try to use multiple measurement methods, such as supervisor or co-worker ratings of CWB, in addition to the types of procedural remedies we employed.

We recognize that phenomena in organizations tend to occur in multilevel networks that are complex. Although we have presented a parsimonious model of key variables, the constructs reflected in our research could be expanded to include both group- and organizational-level constructs, such as measures of group cohesion, organizational culture, or organizational effectiveness. In particular, while we selected organizations from which to sample participants based on an assumed uniform culture within the industry sector (telecommunications), we did not directly test this assumption.

Future studies might also incorporate a small number of additional variables as covariates. These might be stated in terms of (a) states, which could include justice-related contextual variables, such as illegitimate tasks (Omansky et al., 2016); (b) individual differences such as age, which has been shown to moderate the relationship between justice and emotional exhaustion (Brienza and Bobocel, 2017); and (c) as indicated, cultural and demographic factors that impinge on the interrelationships between employees and their managers (Zagenczyk et al., 2015).

In addition, given the prominence of the *low organizational justice with high emotional intelligence* profile we suggest that Machiavellianism, narcissism, and psychopathy be given primary attention as the most prominent socially aversive traits that have been researched in the literature (Paulhus and Williams, 2002). Such traits, frequently discussed as the “dark triad” (Paulhus and Williams, 2002) could potentially moderate the relationships we uncovered between the *low organizational justice with high emotional intelligence* profile and outcomes we noted in this study such as emotional exhaustion. Other elements or types of negative organizational behaviors should also be examined as potential moderators of this profile. This is particularly true of behaviors designed to be injurious to the organization that were not specifically investigated in this study and that seem to covary, such as destructive political behaviors, breaches of confidence, and excessive or inappropriate impression management activities (Griffin and O’Leary-Kelly, 2004; Levy and Tziner, 2011).

To examine the determinants of organizational justice in more detail, we recommend that managers use interview data, which might include performance management conversations, as well as exit-interview data, which could provide a retrospective account of justice perceptions. As part of such studies we would recommend the use of multilevel or mixed-methods research approaches (e.g., Shkoler, 2019) to investigate further the team-level variables in organizational contexts. This would be particularly important for our proposed model, because over 80% of participants in our study did not work in teams. Furthermore, this investigation should be replicated with respondents exhibiting demographic characteristics spanning more evenly over the respective ranges (e.g., more evenly spread over the 26–46 + categories of age).

## Data Availability Statement

The datasets generated for this study are available on request to the corresponding author.

## Ethics Statement

Ethical review and approval was not required for the study on human participants in accordance with the local legislation and institutional requirements. The patients/participants provided their written informed consent to participate in this study.

## Author Contributions

AT: project design and model development. EF: manuscript writing and conceptual framework development. S-KK: data analyses. CV: project design and data analyses. OS: manuscript writing.

## Conflict of Interest

The authors declare that the research was conducted in the absence of any commercial or financial relationships that could be construed as a potential conflict of interest.
